# Glycemic variability and entero-pancreatic hormones signatures after different bariatric surgery procedures: a cross-sectional study

**DOI:** 10.1038/s41366-025-01865-8

**Published:** 2025-08-09

**Authors:** Carolina B. Lobato, Sofia S. Pereira, Marta Guimarães, Bruno Soares, Bolette Hartmann, Mário Nora, Jens J. Holst, Mariana P. Monteiro

**Affiliations:** 1 Endocrine and Metabolic Research, UMIB - Unit for Multidisciplinary Research in Biomedicine, ICBAS - School of Medicine and Biomedical Sciences, University of Porto, Porto, Portugal; 2 ITR - Laboratory for Integrative and Translational Research in Population Health, Porto, Portugal; 3 Department of Biomedical Sciences, Faculty of Health and Medical Sciences, University of Copenhagen, Copenhagen, Denmark; 4 Section of Endocrinology, Department of Medicine, Copenhagen University Hospital – Amager and Hvidovre, Hvidovre, Denmark; 5 Department of General Surgery, Unidade Local de Saúde de Entre o Douro e Vouga, Santa Maria da Feira, Portugal; 6 Department of Medical Oncology, Portuguese Oncology Institute of Porto (IPO-Porto), Porto, Portugal; 7 Novo Nordisk Foundation Center for Basic Metabolic Research, Faculty of Health and Medical Sciences, University of Copenhagen, Copenhagen, Denmark

**Keywords:** Diabetes, Obesity, Translational research, Weight management

## Abstract

**Background/objectives:**

Bariatric surgery changes food handling and entero-pancreatic endocrine dynamics. We aimed at understanding the influence of anatomical reorganization of the gastrointestinal tract induced by metabolic and bariatric surgery (BS) on glycemic variability and the extent to which glycemic variability reflects the underlying entero-pancreatic hormone dynamics.

**Subjects:**

We performed a cross-sectional study on glycemic variability after four different BS procedures in comparison with non-operated matched controls (*n* = 8). The surgical groups were the classic Roux-en-Y gastric bypass (C-RYGB, *n* = 8), a modified long biliopancreatic limb RYGB (M-RYGB, *n* = 7), a single-anastomosis duodeno-ileal bypass with sleeve gastrectomy (SADI-S, *n* = 8) and a biliopancreatic diversion with duodenal switch (BPD-DS, *n* = 7).

**Methods:**

Participants completed 14 days of intermittently scanned continuous glucose monitoring (isCGM). The surgical groups also underwent a mixed-meal test with hormone profiling. Our primary outcome was the mean absolute glucose change (MAG change) in the operated vs non-operated individuals. Additionally, we developed, validated and herein release an automated tool, Gluc4all, for personalized and automated continuous glucose monitoring data analysis, particularly relevant when evaluating the glycemic profile of individuals without diabetes.

**Results:**

All surgical interventions were associated with an increase in the magnitude of postprandial glucose excursions, in anatomy-specific patterns (MAG change was 2.0-fold higher after C-RYGB and M-RYGB and 1.6-fold higher after SADI-S and BPD-DS than in non-operated controls). These isCGM findings matched the postprandial glucose, glucose-dependent insulinotropic peptide (GIP), glucagon-like peptide-1 (GLP-1) and insulin profiles documented in the meal test.

**Conclusions:**

Overall, we show that BS interventions are associated with higher glycemic variability. Moreover, depending on the type of gastrointestinal anatomical reconstruction, BS yields procedure specific glycemic variability patterns. This might be due to faster glucose absorption, impaired amino acid absorption, and/or altered entero-pancreatic hormone profiles, including GLP-1 and insulin secretion.

## Introduction

Bariatric surgery (BS) has been the gold-standard for long-term treatment of severe obesity and related diseases for decades [1–3]. Roux-en-Y gastric bypass (RYGB) is one of the most popular procedures worldwide. A small calibrated gastric pouch is created to limit stomach capacity. This is associated with a reorganization of the small intestine, where three limbs are generated: (1) the duodenum and proximal gut form the *biliopancreatic limb* (BPL) that conveys the biliary and pancreatic juices and does not contact with food; (2) the *alimentary limb* (AL) that comprises a distal segment of the small intestine, which proximally is anastomosed to the gastric pouch and distally anastomosis with the BPL; (3) and lastly, the distal segment of the small intestine that forms the *common channel* (CC; length not measured), where food and biliopancreatic fluids mix and digestion and absorption likely take place. In our center, we perform RYGB with a fixed AL length of 120 cm, and two distinct BPL lengths of 50–100 cm as standard practice, also called classic RYGB (C-RYGB); or with a 200 cm length, as the preferred procedure in patients with diabetes or metabolic syndrome and hereafter designated Metabolic RYGB (M-RYGB) [4].

Over the last decade, our center has also been providing hypoabsorptive procedures to people with a body mass index (BMI) above 45 kg/m^2^. Two procedures are used: the well-established biliopancreatic diversion with duodenal switch (BPD-DS); and the single-anastomosis duodenal-ileal bypass with sleeve gastrectomy (SADI-S), a simplified version of the previous, aimed at reducing surgical time and complications [5]. In both, we perform a vertical sleeve gastrectomy (SG), which keeps the pylorus anatomical integrity, thus potentially allowing regulation of gastric emptying [6].

In BPD-DS, the proximal duodenum is transected approximately 2–3 cm after the pylorus at the level of the gastroduodenal artery. Next, 300 cm small bowels are measured from the caecum and a hand-sewn duodeno-ileal anastomosis is performed, creating one biliopancreatic limb and a common channel. Next, the biliopancreatic limb is anastomosed to the ileum, 100 cm proximal to the ileocecal valve. The latter results in a AL of 200 cm, a CC of 100 cm, and a BPL of variable length (depending on the small intestinal length of the individual) [5].

**Fig. 1:**
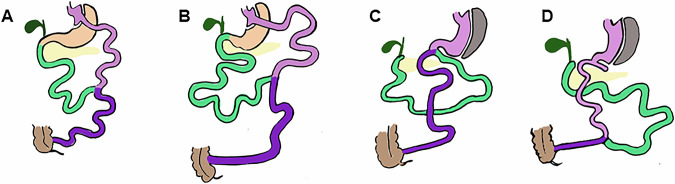
Conceptual representation of the different bariatric procedures. These are classic Roux-en-Y gastric bypass (C-RYGB, **A**), metabolic Roux-en-Y gastric bypass (M-RYGB, **B**), single-anastomosis duodeno-ileal bypass with sleeve gastrectomy (SADI-S, **C**) and biliopancreatic diversion with duodenal switch (BPD-DS, **D**). In pink, the gastric pouches (C-RYGB (**A**) and M-RYGB (**B**)), gastric sleeves (SADI-S (**C**) and BPD-DS (**D**)) and the alimentary limbs (AL), which in C-RYGB (**A**) and M-RYGB (**B**) measure 120 cm and in BPD-DS (**D**) measure 200 cm. In green, the biliopancreatic limbs (BPL) which are formed with the initial portion of the jejuno-ileum. The BPL measures 50–100 cm in C-RYGB (**A**), 200 cm in M-RYGB (**B**) and variable lengths in SADI-S (**C**) and BPD-DS (**D**). Lastly, in purple, the common channel (CC), which has a variable length in C-RYGB (**A**) and in M-RYGB (**B**) and fixed length of 300 cm in SADI-S (**C**) and 100 cm in BPD-DS (**D**).

In SADI-S, only the proximal duodenal transection followed by anastomosis to small bowels is performed, precisely as previously described. This results in the creation of a CC of fixed length (300 cm) and a BPL of variable length. Thus, the CC, responsible for digestion and absorption, is shorter in the BPD-DS than in the SADI-S [7] (Fig. 1).

While these BS interventions are very effective at guaranteeing long-term glycemic control and type 2 diabetes remission [2], they accelerate food transit, change entero-pancreatic hormone dynamics and increase overall glycemic variability. These have been linked with complications, such as micronutrient deficiencies, dumping syndrome, and postprandial hypoglycemia [8, 9]. Whether or not the anatomical reconstruction after surgery modulates glycemic variability profiles differently according to food handling and/or entero-pancreatic hormone responses is not yet clear.

The aim of this study is to understand the role of the anatomical reorganization induced by BS on glycemic variability and to elucidate the extent to which glycemic variability reflects the underlying entero-pancreatic hormone dynamics.

## Materials/subjects and methods

### Setting

We performed a cross-sectional study at a national reference center, which holds extensive experience performing RYGB, SADI-S and BPD-DS interventions. This study was approved by the Institutional Ethics Committee and conduced in compliance with the ethical principles outlined in the Declaration of Helsinki and with the GDPR regulations.

### Study participants

A single researcher (M.G.) recruited the surgical participants. Firstly, we identified and invited potential participants submitted to the two kinds of RYGB with known weight stability and acceptable glycemic control from our retrospective databases. We next recruited patients submitted to SADI-S and BPD-DS with post-operative similar features to the groups submitted to RYGB. The two hypoabsorptive procedures were more recently introduced at our center and therefore there were no patients with the same follow-up duration as for RYGB available at the time of the recruitment for this study. Instead, patients submitted to SADI-S and BPD-DS were recruited at their 12- or 24-month post-surgery follow-up outpatient visits and invited into the study only if they were found to be weight stable at that time. Lastly, we invited weight-stable subjects without diabetes and without history of gastrointestinal surgery, with similar age, weight and sex distribution as the surgical groups (control group). All subjects signed informed consent. If fulfilling all inclusion and exclusion criteria, they were enrolled in the study (Supplementary Table 1).

### Study protocol

During a baseline visit, we reviewed the participants’ medical and surgical history, performed anthropometric measurements, biochemical blood tests and scheduled the meal test study visit.

We instructed participants to fast for at least 8 hours and to present at 8 am for a mixed meal tolerance test (MMTT). We placed a venous antecubital cannula and collected two baseline blood samples. Afterwards, participants drank a Fresubin Energy Drink® (35% fat, 50% carbohydrates, 15% protein; 200 mL; 7.8–12.6 g of sugars) over a period of 15 minutes. We sampled blood at 15, 30, 45, 60, 90 and 120 minutes from meal start. We monitored glycemia, blood pressure and heart rate at the same timepoints. Afterwards, we served a meal and ensured euglycemia before discharge.

In parallel, we started the participants on 14-days of open-label intermittently scanned continuous glucose monitoring (isCGM) using Libre 1 (Abbott®).

The control group only underwent the screening visit and the 14-days isCGM.

### Samples handling and biochemical measurements

We sampled blood into EDTA tubes that were centrifuged at 4 *°*C, 2500 G, for 12 minutes. Afterwards, we aliquoted plasma and froze it at −20 *°*C, pending batch analysis.

We measured blood glucose directly from the venous cannula using a glucometer (Precision Neo, Abbott, USA); plasma insulin and C-peptide through electrochemiluminescence sandwich immunoassay (ECLIA) using Cobas 8000, e602 (Roche Diagnostics, USA); total amino acids (TAA) as described [10]; glucagon, glucagon-like peptide-1 (GLP-1), glucose-dependent insulinotropic peptide (GIP) and neurotensin through radioimmunoassay (antibodies 4305, 89390, 80867 and 3D97) [11–14] in two batches. For radioimmunoassays, intra-assay CV was below 10%, and internal controls for the two batches were similar.

We previously published some of these data with a focus solely on the entero-pancreatic hormone profiling of each BS intervention [4, 5]. Herein we focus on glycemic variability tendencies and use the entero-pancreatic hormone dynamics to further elucidate these.

### isCGM data analysis

We previously described in detail our isCGM data analysis protocol [15]. In brief, we excluded data collected during the first 48 hours to ensure data accuracy, and manually analyzed the clean dataset afterwards. We computed summary metrics to objectively evaluate the >1000 isCGM records retrieved per participant. Such metrics include time in range and deviations, central tendency measures (mean, standard deviation [SD] and percentiles), metrics that target the rate (mean absolute glucose change [MAG change], continuous overlapping net glycemic action [CONGA1], mean of daily differences [MODD], coefficient of variation [CV]) and magnitude (CV, maximum, and average daily risk ratio [adjusted; ADRR_FGM_GT]) of glucose changes, which relate to a risk of both hypo and hyperglycemia (low blood glucose index [adjusted; LBGI_FGM_GT] and high blood glucose index [adjusted; HBGI_FGM_GT]). Each dataset was independently analyzed by at least two researchers (B.M.S., C.B.L., S.S.P.).

Using Visual Basic for Applications, we developed an automated tool to optimize continuous glucose monitoring (CGM) data analysis. The tool uses Excel® as interface, and was cross-validated against the manual isCGM data analysis of the 38 isCGM files included in this study. We established the accuracy of the automated tool using percentage differences and Student’s t-tests (Gluc4all, version 1.0.0; validation information and display as **Supplementary material**). This tool is herein made available for academic research use (bottom of page: Endocrine and Metabolic Research - ICBAS).

### Meal test data analysis

We took two parallel approaches to analyze the meal test data. One targeted the immediate postprandial response, for which the area under the curve (AUC) up to peak was considered (Supplementary Table 2); and another one encompassed the overall response to a meal up to 2 hours (AUC 0’-120’).

We evaluated β-cell function and insulin resistance using surrogate measures known as the homeostasis model assessment indexes (HOMA2-B and HOMA2-IR; HOMA Calculator version 2.2.3, http://www.dtu.ox.ac.uk, accessed April 2018).

We calculated excess BMI loss (%EBMIL) as the ratio of the BMI lost since surgery over the BMI required to achieve a target BMI of 25 kg/m^2^.

We calculated estimated percentage of glycated hemoglobin after the mean glucose values on FGM, as previously established [16].

### Statistical analysis details

We assessed the normality of data distribution by inspection of Q-Q plots and using the Shapiro-Wilk test. In case of disagreement, a second researcher inspected the Q-Q plot. Preference to normal testing was given (one-way ANOVA with multiple testing). We log transformed skewed data, except when the dataset included zeros. We analyzed non-parametric data using Kruskal-Wallis tests with multiple comparisons. We compared each surgical group with the non-operated controls and, in parallel, the surgical groups were compared among themselves. We adjusted *p* values for multiple comparisons (*n* = 4 comparing surgeries vs controls; and *n* = 6 comparing surgeries).

We additionally performed Spearman correlations between metrics of glycemic variability in the isCGM and in the meal test and between glycemic variability in isCGM and the hormonal responses during the meal test.

We analyzed the data using GraphPad® Prism® 10.2.2, and Microsoft® Excel® 365.

### Pre-specified study outcomes

The primary outcome of this study was glycemic variability, evaluated as the mean absolute glucose change (MAG change) in the operated individuals vs non-operated controls. The secondary outcome of special interest was the MAG change differences between the surgical groups. Other exploratory outcomes targeted the entero-pancreatic hormone dynamics and thus we focused on comparing the postprandial secretion (up to peak) of GLP-1 and of C-peptide in the different surgical groups.

## Results

Table 1 describes the baseline characteristics of the participants. At assessment, the BMI, the %EBMIL, insulin resistance and β-cell function of all participant groups were similar. Time since surgery was higher after the RYGB procedures, and total weight loss (%TWL) was higher after the hypoabsorptive surgeries (Table 1).

**Table 1:** Characterization of the study population.

Variable	C-RYGB	M-RYGB	SADI-S	BPD-DS	Control
Sex (M/F)	1/7	1/6	1/7	3/4	2/6
Age (years)	50.9 [45.2−56.6]	51.4 [44.9−58.0]	47.4 [42.4−52.5]	40.4 [29.7−51.1]	47.7 [45.1−50.4]
Weight (kg)	74.1 [65.6–82.7]	68.4 [59.1–77.7]	76.8 [71.0–82.5]	77.2 [63.5–90.8]	81.9 [74.1–89.6]
BMI (kg/m^2^)	28.3 [26.4−30.3]	26.6 [23.9−29.3]	29.9 [26.7−33.1]	28.0 [25.3−30.7]	29.0 [25.7−32.2]
A1c (%)	5.5 (5.2–5.8)	5.3 (5.0–5.6)	4.8 (4.2–5.0)	4.9 (4.0–5.0)	–
eA1c (%)	4.9 [4.6–5.1]	4.9 [4.7–5.1]	4.8 [4.4–5.2]	4.7 [4.4–5.0]	5.0 [4.7–5.3]
HOMA2-IR	0.86 (0.78–1.03)	0.64 (0.52–0.89)	0.58 (0.36–0.75)	0.66 (0.39–0.85)	–
HOMA2-β	90.6 (75.2–101.8)	65.6 (49.4–85.3)	69.8 (43.2–84.4)	67.9 (54.8–84.7)	–
Time since surgery (years)	5.2 (3.0–7.6)	5.7 (3.2–6.0)	2.4 (2.1–2.6)	2.6 (2.2–2.7)	-
Weight pre op (kg)	109.1 [92.2–126.1]	102.3 [90.7–113.9]	134.9 [117.0–152.8]	141.4 [119.1–163.7]	–
%TWL	31.3 [24.9–37.7]	33.0 [26.7–39.3]	42.4 [37.2–47.5]	45.5 [42.5–48.5]	–
BMI pre op	41.7 (38.5–43.6)	39.4 (38.3–41.2)	50.5 (49.3–55.7)	50.7 (47.3–54.7)	–
%EBMIL	79.0 (66.5–89.8)	86.2 (73.4–112.5)	80.9 (70.9–94.0)	89.8 (80.1–97.9)	–
A1c pre op	5.2 [4.7–5.6]	5.9 [5.4–6.4]	5.6 [5.1–6.0]	5.4 [4.9–6.0]	-

Demographic and anthropometric features of the study participants distributed per study group (classic gastric bypass [C-RYGB, *n* = 8], metabolic gastric bypass [M-RYGB, *n* = 7], single anastomosis duodenal-ileal bypass with gastric sleeve [SADI-S, *n* = 8], biliopancreatic diversion with gastric sleeve [BPD-DS, *n* = 7] and non-operated matched individuals [Control, *n* = 8]. Data is presented as mean [95% confidence interval of the mean] or median (interquartile range).

*BMI* body mass index, *A1c* glycated haemoglobin, *HOMA2-B* homeostasis model assessment of β-cell function, *HOMA2-IR* homeostasis model assessment of insulin resistance, *eA1c (%)* estimated percentage of glycated haemoglobin, *%EBMIL* percentage of excess body mass index loss, *%TWL* percentage of total weight loss.

isCGM capture rates were similar between the study groups and above the 70% cutoff recommended by ADA [17] (Table 2).

**Table 2:** isCGM glycaemic variability data.

Variable	C-RYGB	M-RYGB	SADI-S	BPD-DS	Control
Duration (days)	11.9 (11.9–11.9)	11.8 (11.7–11.9)	11.8 (9.8–11.9)	11.9 (11.7–11.9)	11.9 (11.9–12.0)
Data capture rate (%)	94.4 (86.4–98.7)	96.6 (89.8–99.6)	94.6 (80.2–98.9)	92.5 (79.0–96.4)	93.4 (84.5–97.5)
TIR (%)	80.3 (58.3–86.8)	80.3 (76.2–90.8)	88.9 (64.0–94.8)	89.7 (78.9–96.1)	97.6 (94.9–99.8)
Time < 3.9 mmol/L (%)	14.2 (3.5–36.5)	11.7 (3.2–17.1)	5.9 (1.1–35.0)	10.2 (1.0–21.0)	0.0 (0.0–4.3)
Time < 3.0 mmol/L (%)	0.7 (0.2–2.3)	0.4 (0.0–1.4)	0.4 (0.0–4.1)	0.1 (0.0–2.1)	0.0 (0.0–0.0)
Time > 7.8 mmol/L (%)	6.5 (4.1–9.4)	5.1 (3.3–9.5)	2.3 (0.3–4.5)	0.9 (0.1–3.9)	0.3 (0.0–1.1)
P10 (mmol/L)	3.7 [3.4–4.0]	3.8 [3.6–4.1]	3.8 [3.4–4.3]	3.9 [3.4–4.4]	4.6 [4.1–5.1]
P25 (mmol/L)	4.1 [3.7–4.4]	4.2 [4.0–4.4]	4.2 [3.7–4.7]	4.2 [3.8–4.7]	4.8 [4.4–5.3]
P50 (mmol/L)	4.6 [4.2–5.0]	4.7 [4.5–4.9]	4.8 [4.2–5.3]	4.6 [4.2–5.1]	5.2 [4.8–5.6]
P75 (mmol/L)	5.6 [5.1–6.1]	5.9 [5.5–6.4]	5.6 [5.0–6.3]	5.5 [5.0–6.1]	5.7 [5.3–6.1]
P90 (mmol/L)	7.5 [7.0–7.9]	7.7 [6.8–8.5]	6.7 [5.8–7.5]	6.5 [5.9–7.2]	6.4 [5.9–6.9]
Maximum (mmol/L)	13.0 [11.2–14.7]	12.3 [10.0–14.6]	9.8 [8.0–11.6]	9.2 [8.2–10.1]	9.0 [7.8–10.2]
LBGI_FGM_GT	5.1 (2.7–8.9)	4.0 (2.3–5.0)	3.2 (1.4–9.3)	4.6 (1.7–6.2)	0.5 (0.1–2.4)
HBGI_FGM_GT	2.2 (1.4–2.6)	1.8 (1.1–2.4)	1.2 (0.3–1.5)	0.5 (0.4–1.6)	0.5 (0.4–1.0)
MAG change (mmol/L × h^−1^)	2.4 [2.2–2.7]	2.2 [1.7–2.6]	1.6 [1.2–2.0]	1.6 [1.3–1.9]	1.0 [0.8–1.2]
CONGA1	2.3 (1.9–2.4)	2.0 (1.6–2.2)	1.4 (1.0–1.7)	1.3 (1.2–1.4)	0.8 (0.7–1.0)
MODD	1.3 [1.2–1.5]	1.2 [1.0–1.5]	0.9 [0.7–1.1]	0.9 [0.7–1.0]	0.6 [0.5–0.8]
ADRR_FGM_GT	62.2 [41.9–82.5]	48.2 [31.5–65.0]	35.9 [21.9–50.0]	29.6 [22.2–36.9]	15.7 [7.8–23.6]
Mean	5.2 [4.8–5.5]	5.3 [4.9–5.6]	5.0 [4.5–5.6]	5.0 [4.5–5.4]	5.4 [4.9–5.8]
SD	1.7 (1.5–1.9)	1.5 (1.3–1.8)	1.2 (0.9–1.5)	1.0 (0.9–1.2)	0.7 (0.6–0.9)
CV (%)	34.4 (29.3–37.2)	28.7 (27.2–33.1)	24.7 (17.4–26.7)	20.3 (18.7–25.6)	13.0 (11.8–16.9)

Data from intermittently scanned continuous glucose monitoring (isCGM) of the study participants distributed per study group (classic gastric bypass [C-RYGB, *n* = 8], metabolic gastric bypass [M-RYGB, *n* = 7], single anastomosis duodenal-ileal bypass with gastric sleeve [SADI-S, *n* = 8], biliopancreatic diversion with gastric sleeve [BPD-DS, *n* = 7] and non-operated matched individuals [Control, *n* = 8]. Data is presented as mean [95% confidence interval of the mean] or median (interquartile range).

*TIR* time in range, *P* percentile, *LBGI*_FGM_GT low blood glucose index (adjusted), *HBGI*_FGM_GT high blood glucose index (adjusted), *MAG change* mean absolute glucose change, *CONGA1* continuous overlapping net glycemic action, *MODD* mean of daily differences, *ADRR*_FGM_GT average daily risk ratio (adjusted), *SD* standard deviation, *CV* coefficient of variation.

**Fig. 2:**
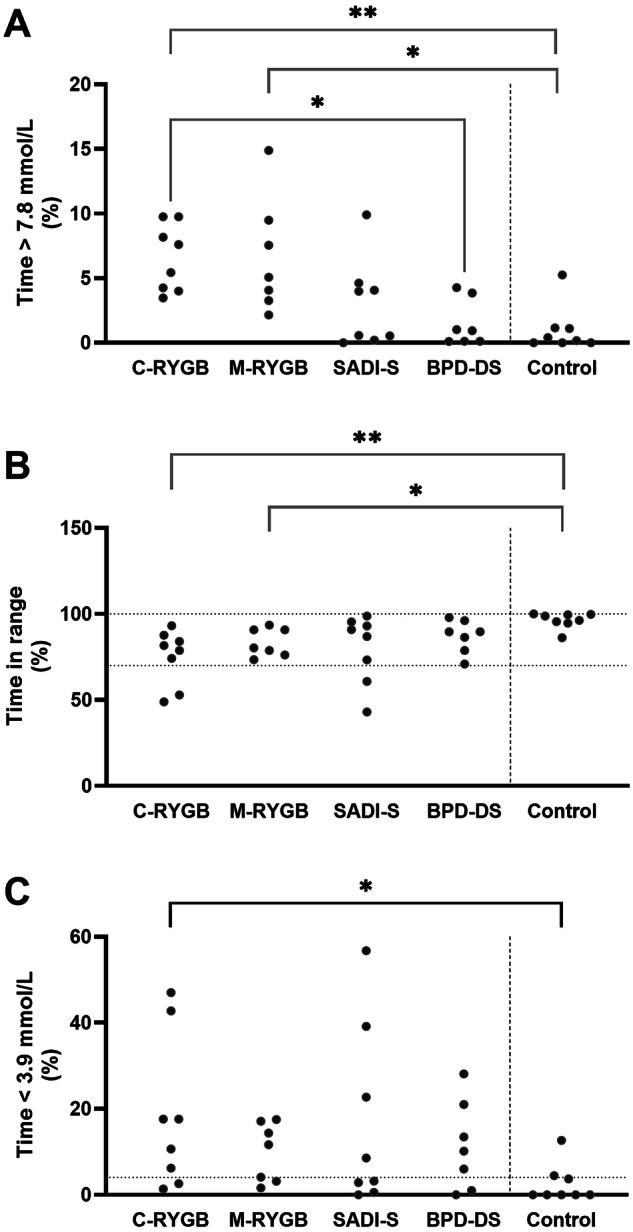
Overview of isCGM profiles. Time above range (% time 7.8 mmol/L, **A**), in range (% time 3.9–7.8 mmol/L, **B**) and in hypoglycemia (% time 3.9 mmol/L, **C**) during isCGM. Study participants are distributed per study group (classic gastric bypass [C-RYGB, *n* = 8], metabolic gastric bypass [M-RYGB, *n* = 7], single anastomosis duodenal-ileal bypass with gastric sleeve [SADI-S, *n* = 8], biliopancreatic diversion with gastric sleeve [BPD-DS, *n* = 7] and non-operated matched individuals [Control, *n* = 8]. The individuals submitted to RYGB had greater time 7.8 mmol/L and lesser time in range than the non-operated controls. Those submitted to CRYGB were also more time 3.9 mmol/L than the control. The dashed lines represent the target ranges for continuous glucose monitoring according to the ADA consensus [17] (time in range between 70−100%, and time below range lesser than 4% of the monitored time). *p < 0.05; **p < 0.01.

The surgical groups spent overall less time in range and longer time in hypoglycemia than non-operated controls. Yet, no differences stand out between participants submitted to different procedures: while RYGB operated individuals displayed overall higher percentages of time in hypoglycemia, there was a bimodal tendency for hypoglycemia in the participants submitted to hypoabsorptive procedures: one third of participants were less than 4% of time under mild hypoglycemia (four participants submitted to SADI-S and two to BPD-DS glucose < 3.9 mmol/L; target settled at ADA consensus [17]) whereas one third experienced hypoglycemia more than 20% of the time (three submitted to SADI-S and two to BPD-DS) (Fig. 2).

The non-operated individuals had less than 1% of time under severe hypoglycemia (<3.0 mmol/L), which is a target settled at ADA consensus [17] (one-sample Wilcoxon t-test against 1: *p* < 0.01). This was not true for the operated groups (*p* > 0.05) (Fig. 2).

In comparison with controls, percentiles 10 and 25 (P10 and P25) of the isCGM data were lower in each surgical group. Individuals submitted to C-RYGB displayed an increased risk of hypoglycemia (LBGI_FGM_GT). Only participants submitted to the two kinds of RYGB interventions had a significantly greater tendency to hyperglycemia (eg. time above glucose > 7.8 mmol/L, percentile 90 (P90) and maximum; Table 2).

**Fig. 3:**
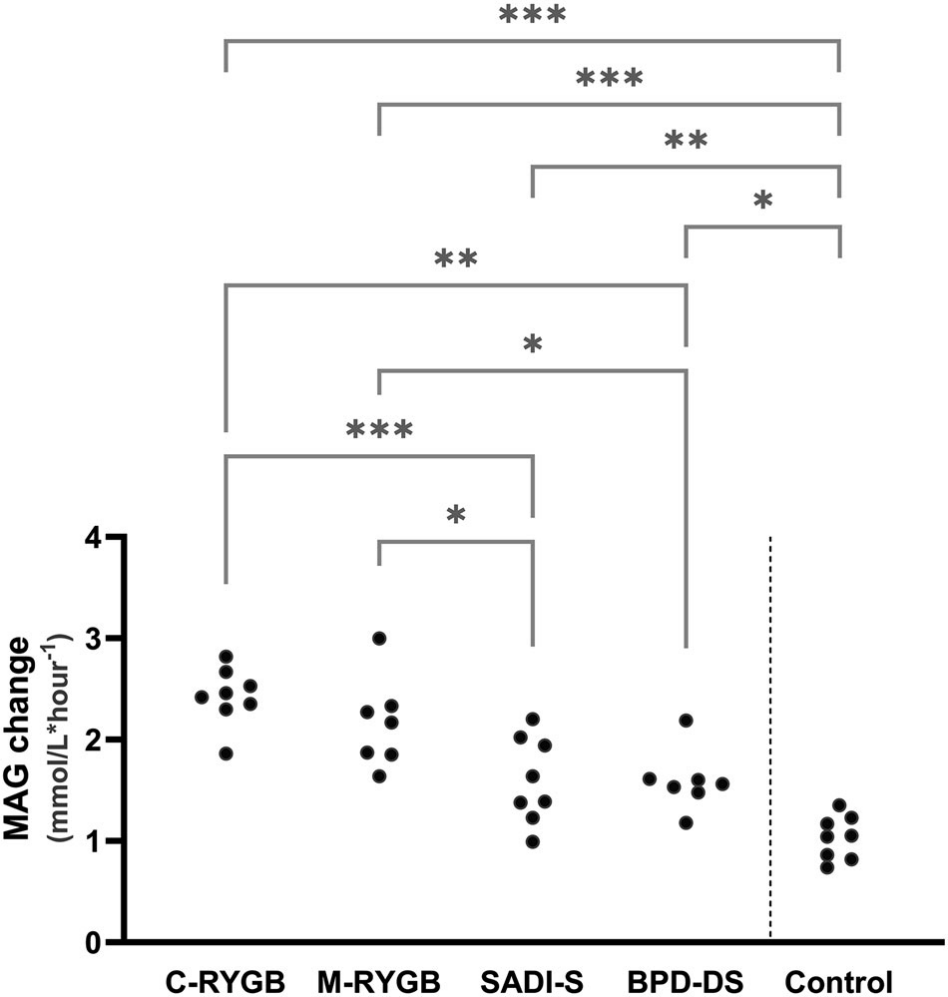
Mean absolute glucose change (MAG change) during isCGM, as a measure of glycemic variability. Study participants are distributed per study group (classic gastric bypass [C-RYGB, *n* = 8], metabolic gastric bypass [M-RYGB, *n* = 7], single anastomosis duodenal-ileal bypass with gastric sleeve [SADI-S, *n* = 8], biliopancreatic diversion with gastric sleeve [BPD-DS, *n* = 7] and non-operated matched individuals [Control, *n* = 8]. MAG change is presented as a measure of glycemic variability. All surgical groups display increased glycemic variability in comparison with the non-operated controls (primary endpoint). Glycemic variability is greater in RYGB groups vs hypoabsortive procedures (SADI-S and BPD-DS) (secondary outcome of special interest). *p < 0.05; **p < 0.01; ***p < 0.001.

Glycemic variability metrics which target the rate of glucose changes (MAG change and CONGA1), the inter-daily differences between glucose patterns (MODD) and the overall dispersion of glucose profiles (ADRR_FGM_GT and CV) failed to disclose any differences in the isCGM glucose dynamics between the two kinds of RYGB and between the two hypoabsorptive procedures. Nevertheless, all surgical interventions led to an outstanding increase in glycemic variability, most pronounced in those submitted to RYGB, followed by those who underwent hypoabsorptive interventions, both by comparison with the non-operated individuals but also with one another (Fig. 3). The CV had the least power to detect such differences (Table 2).

These isCGM findings perfectly match the postprandial glucose profiles documented in the meal test. The greatest magnitude of glycemic variability documented on isCGM achieved after C-RYGB is also documented by higher glucose peak and nadir and by the maximum-to-minimum glucose ratio during the test (MMGR) (Table 3).

**Table 3:** Entero-pancreatic dynamics following a mixed meal.

	C-RYGB	M-RYGB	SADI-S	BPD-DS
**Glucose**				
Fasted (mmol/L)	4.5 [4.2–4.8]	4.6 [4.3–4.8]	4.4 [4.0–4.7]	4.4 [4.1–4.7]
Peak (mmol/L)	8.8 (8.5–9.4)	8.8 (7.2–9.2)	7.0 (6.6–8.2)	5.4 (5.2–6.7)
Nadir (mmol/L)	2.8 [2.3–3.4]	3.4 [2.9–3.8]	3.7 [3.1–4.2]	3.0 [2.5–3.6]
MMGR	3.3 [2.6–3.9]	2.5 [2.1–3.0]	2.0 [1.8–2.2]	2.0 [1.6–2.3]
tAUC (0’–30’) (mmol/L × min)	208 [194–222]	190 [177–204]	168 [157–180]	149 [136–162]
tAUC (0’–120’) (mmol/L × min)	637 [587–687]	683 [596–770]	620 [536–705]	526 [441–611]
**TAA**				
Fasted (μmol/L)	1627 [1389–1864]	1336 [994–1678]	1161 [867–1455]	1042 [884–1201]
tAUC (0’–45’) (mmol/L × min)	114.2 [101.2–127.2]	99.0 [75.7–122.3]	93.4 [72.0–114.4]	59.2 [43.4–75.0]
tAUC (0’–120’) (mmol/L × min)	268.4 (261.5–294.8)	262.8 (234.9–295.4)	245.4 (231.9–269.6)	123.6 (105.5–204.5)
**Heart rate**				
Fasted (bpm)	62 (55–71)	60 (58–76)	64 (57–73)	67 (55–80)
tAUC (0’–30’) (bpm × min)	2436 (2247–2715)	2119 (2006–2490)	2051 (1721–2085)	2040 (1767–2399)
tAUC (0’–120’)(bpm × min)	9306 (8795–10204)	8486 (7886–10035)	8419 (7785–9053)	8250 (7616–9419)
**Insulin**				
Fasted (pmol/L)	45.7 (41.9–54.5)	33.8 (26.7–47.3)	30.8 (19.0–40.4)	35.2 (21.0–45.3)
tAUC (0’–45’) (nmol/L × min)	64.6 (34.7–78.7)	26.2 (12.3–29.3)	22.5 (15.8–35.4)	17.3 (10.8–29.2)
tAUC (0’–120’) (nmol/L × min)	94.8 [65.2–124.3]	54.3 [37.9–70.7]	44.7 [31.7–57.8]	37.5 [25.5–49.5]
**C-peptide**				
Fasted (pmol/L)	578.1 (499.1–615.8)	478.3 (373.9–580.6)	399.0 (333.8–532.1)	330.3 (309.3–495.9)
tAUC (0’–45’) (nmol/L × min)	154.3 (99.5–172.3)	84.9 (61.2–91.1)	66.5 (60.1–90.4)	59.9 (44.4–70.8)
tAUC (0’–120’) (nmol/L × min)	322.6 (241.2–380.5)	227.5 (186.3–297.6)	176.9 (160.8–242.2)	167.0 (145.4–194.3)
**Glucagon**				
tAUC (0’–30’) (nmol/L × min)	322.7 [257.3–388.2]	343.9 [198.8–489.1]	414.9 [285.4–544.4]	332.9 [271.8–393.9]
tAUC (0’–120’) (nmol/L × min)	1428 [1144–1713]	1746 [1193–2298]	1650 [1194–2106]	1285 [901.6–1668]
**Total GIP**				
tAUC (0’–30’) (pmol/L × min)	1632 (1536–2340)	922 (742–1103)	540 (257–938)	390 (236–841)
tAUC (0’–120’) (pmol/L × min)	5018 (4281–6925)	3420 (2498–3668)	1628 (864–4389)	1418 (1181–3389)
**Total GLP-1**				
tAUC (0’–30’) (pmol/L × min)	2869 (2239–3358)	2633 (2048–2970)	1828 (1562–3048)	1527 (1267–2676)
tAUC (0’–120’) (pmol/L x min)	7898 (6205–10142)	13388 (7808–14670)	6612 (5948–9485)	5993 (4576–9117)
**Neurotensin**				
tAUC (0’–30’) (pmol/L × min)	3004 (2014–5546)	3900 (2018–5153)	2012 (1159–4013)	2455 (2150–3181)
tAUC (0’–120’) (pmol/L × min)	12481 (7707–16622)	17025 (10553–21638)	9360 (6176–15630)	14010 (12110–15488)

Entero-pancreatic hormone profile during the meal test distributed per study group (classic gastric bypass [C-RYGB, *n* = 8], metabolic gastric bypass [M-RYGB, *n* = 7], single anastomosis duodenal-ileal bypass with gastric sleeve [SADI-S, *n* = 8] and biliopancreatic diversion with gastric sleeve [BPD-DS, *n* = 7]. Data are presented as mean [95% confidence interval of the mean] or median (interquartile range).

*tAUC* total area under the curve, *TAA* total amino acids, *GIP* glucose-dependent insulinotropic polypeptide, *GLP-1* glucagon-like peptide-1.

There were strong associations between metrics of glycemic variability determined using the isCGM data and the glycemic excursions during the meal test. In detail, standard deviation (SD), maximum, time above glucose > 7.8 mmol/L, HBGI_FGM_GT, MAG change, CONGA, MODD and ADRR_FGM_GT during isCGM correlates with MMGR, peak, glucose and TAA during the MMTT. We found no correlation with nadir glucose on MMTT (Supplementary Table 3).

During the meal test, the rate of appearance of glucose, but especially of amino acids (AA) was lower in individuals submitted to BPD-DS than any other intervention, with delayed postprandial peaks and smaller postprandial excursions (Table 3).

The postprandial secretion of insulin and its co-secreted sub-product C-peptide was more pronounced in individuals submitted to C-RYGB, intermediate after M-RYGB and SADI-S procedures, and less noticeable after BPD-DS. This response was preceded by earlier distinct postprandial secretion of GIP, in the exact same pattern (Table 3).

The postprandial GLP-1 response was greater after both RYGB procedures than after the hypoabsorptive procedures. The adrenergic response to the meal, assessed by the increase in heart rate, and the levels of glucagon, and neurotensin were similar across the surgical groups (Table 3).

Glycemic variability on isCGM (as measured by MAG change) was strongly correlated with the excursions for glucose, TAA, insulin, C-peptide and GIP during the MMTT. There was, however, no association with glucagon or neurotensin excursions (Supplementary Table 4).

The cross-validation of Gluc4all v. 1.0.0. against manual FGM data analysis revealed no differences between the two methods (Supplementary Material).

Statistical analysis details are provided in the Supplementary Material (Supplementary Tables 5−7).

## Discussion

We describe a cross-sectional study of glycemic variability after different BS, by mutual comparisons, and against a matched non-operated group. We show that increased glycemic variability after BS is linked with an increased rate and magnitude of postprandial glucose excursions towards both hyper- and hypoglycemia. This is likely to be due to different rates of intestinal entry, and differences in postprandial handling of nutrients and entero-pancreatic hormone secretion, with highlight to faster glucose absorption, impaired amino acid absorption, and/or altered entero-pancreatic hormone profiles. Some people submitted to hypoabsorptive procedures frequently experience events of mild and severe hypoglycemia. Finally, there is a higher tendency for glucose ranges towards lower values and hypoglycemia after BS, in likely association with the altered entero-pancreatic endocrine dynamics. The long-term clinical impact of these findings remains to be established, but the tool herein released for CGM data analysis (Gluc4all) will enable reproduction of our findings on glycemic variability and entero-pancreatic hormone dynamics after bariatric surgery by other research groups.

One of the major strengths of this study is the systematic approach to distinct anatomical reconstructions during BS, which enables us to draw inferences about the impact of the anatomical manipulation/reorganization in glycemic variability. The average length of the human small intestine is 5.3 m although this is variable [18]. Bearing this in mind, C-RYGB and BPD-DS stand out as opposite extremes of the BS anatomical reconstructions spectrum: on the one hand, the C-RYGB population where intestinal nutrient entry is not regulated and the bypassed intestinal portion is the shortest; on the other hand, the BPD-DS population where gastric emptying regulation is expectedly preserved but the common limb for absorption is the shortest. The two other procedures – SADI-S and M-RYGB – achieve an intestinal bypass length that is intermediate (this is, longer than in the C-RYGB and shorter than in the BPD-DS). Yet, these differ critically in the stomach anatomical reconstruction, as a gastric sleeve is created in the SADI-S and a calibrated gastric pouch is created in the M-RYGB, with preservation or not of the pylorus integrity and thus potentially enabling or not the physiological regulation of gastric emptying rate, respectively.

Does the increased glycemic variability reflect differences in food ingestion? Or can accelerated intestinal nutrient entry be accounted for the increased glycemic variability? Can a longer portion of intestine bypassed be linked with altered entero-pancreatic hormone profile, increased glycemic variability and higher risk of hypoglycemia? Or might bypassing too much intestine impair digestion and/or absorption, and lead to lesser glycemic variability but also to a high tendency of hypoglycemia? These are a few of the questions our study has enabled us to address.

We explored whether food intake is linked with glycemic variability. Acknowledging that collecting free-living dietary intake information is reliant on self-report and thus prone to biases, we chose to ask the participants to have the same meal during test settings and assess whether that prompted a similar glycemic variability profile despite the differences documented in free-living settings.

We relied on the postprandial glucose and AA profiles as surrogates of intestinal nutrient entry, digestion and absorption. This is a reasonable approach as evidenced by the postprandial profiling of absorbed and endogenous glucose and phenylalanine radiolabeled after BS [6]. Such approach does not account for lipids absorption, which is also accelerated but less rapidly and often incompletely absorbed [19, 20] and, therefore, unlikely to play a major role in the early postprandial entero-pancreatic hormone responses.

The rate of gastric emptying has a great influence on the rate of glucose absorption, because glucose is easily absorbed anywhere in the gut [21]. Indeed, the limiting factor for glucose absorption seems to be gastric emptying rate rather than the extent of intestinal bypass, since a rapid postprandial glucose excursion is observed after both RYGB interventions; in contrast to the slightly delayed absorption in participants submitted to hypoabsorptive procedures, in whom gastric emptying is likely retarded through pylorus preservation. Of notice, in an attempt of delaying intestinal entry, the RYGB gastric pouch outlet can be calibrated, as it is our standard practice; while some patients with SG can experience biliary reflux, which flags pyloric dysfunction despite the preserved anatomical integrity [22].

This conclusion is supported by the metrics of glycemic variability on isCGM, which followed the exact same pattern: distinct between operated and non-operated individuals, and between RYGB and hypoabsorptive procedures, without too much contrast between interventions that achieve similar rate of intestinal nutrient entry profile but distinct intestinal bypassed portions. MAG change – our primary endpoint – stands out as the most reliable marker of glycemic variability. In contrast, CV fails to accurately document these tendencies. ADA recommends a target of CV ≤ 36% to reflect good glycemic variability control [17]. However, mean, SD and CV assume data normal distribution, which does not reflect the nature of isCGM data, particularly when the percentage of hypoglycemia is not negligible.

The rate of TAA increase in circulation reflects protein digestion and absorption [6]. We document a markedly slower appearance of TAA in BPD-DS vs all other surgical groups, that aligns with the impaired digestion of proteins and/or absorption of AA formerly documented [7].

One striking finding in our study was the bimodal pattern of hypoglycemia among the participants submitted to hypoabsorptive procedures. While some participants had quite innocent glycemic patterns with not so many low glucose records, we recorded regular and long lasting periods of low glucose in ~30% of the participants submitted to SADI-S and BPD-DS. This was previously documented [23–25], without any clear pathophysiological explanation. The participants with this greater tendency for hypoglycemia were investigated more than 2 years after surgery, were weight stable, and had a similar BMI at time of assessment. A catabolic state with depletion of glycogen is, thus, unlikely. We show that TAA tend to be lower after hypoabsorptive procedures, both fasting and postprandially. We have also previously documented lower essential AA levels in individuals submitted to BPD-DS, vs SADI-S [26]. We know that some AA are strong stimuli for glucagon and GLP-1 secretion, but mostly if absorbed [27].

The faster nutrient absorption after BS is parallelled by an increase in enteric insulinotropic hormone secretion and increased insulin secretion [21]. An overshot in these responses and/or an impairment of the counterregulatory insulinostatic response are likely involved in the increased risk of hypoglycemia after BS that we document.

GIP secretion is believed to be triggered mostly by glucose and fat, and lesser by protein [28]. GIP secreting K-cells are also predominantly located in the upper gut [29]. The much higher levels of GIP secreted postprandially after C-RYGB vs the other bariatric interventions are consistent with this anatomical feature. Interestingly, the GIP secretion also seems to be delayed in the participants that have had hypoabsorptive procedures with pyloric integrity and thus relatively retarded gastric emptying and nutrient absorption. Similar findings have been previously reported by comparing RYGB and SG postprandial GIP profiles [6].

GLP-1 secretion is elicited by glucose and nutrients entry and absorption into the intestinal lumen [28]. GLP-1 is secreted by the L-cells which are more abundant distally in the gut [29]. A tendency for greater postprandial GLP-1 secretion after M-RYGB is observed. This is consistent with both the distal intestinal bypass and the rapid nutrient intestinal entry achieved by this surgery. Of notice, GLP-1 secretion seems to be synchronous in all four procedures and to match perfectly glucose appearance rate, which points towards a predominance of glucose-induced GLP-1 secretion [30].

It is puzzling that there are no differences in glucagon secretion despite the distinct glycemic variability signatures and distinct insulin secretion profiles. Glucagon secretion seems to be acutely increased in the postprandial period after SG and RYGB, but fails to rise as glucose decreases in a later postprandial stage [6].

If it is true that GLP-1 does not increase the risk of hypoglycemia in non-operated individuals [31], that does not seem to be true after RYGB and other operations [32]. It is plausible that the neuroendocrine regulation of the pancreatic response to hypoglycemia might be impaired by the manipulation of the GI tract and/or interference with pancreatic innervation. Some evidence starts to emerge supporting an impairment of parasympathetic activity in people submitted SG or RYGB [33]. It is still speculative whether this might be the missing piece for understanding the increased risk of hypoglycemia, the deficit of counter-regulatory glucagon secretion and the great glycemic variability after BS.

The relative contribution of GIP and GLP-1 to the incretin effect observed in healthy individuals and after RYGB and SG after an oral glucose tolerance test and a mixed meal has been described. In non-operated individuals, the incretin effect is attributable mostly to GIP, secondly to GLP-1, but also to glucose and other enteroendocrine factors [34, 35]. GIP and GLP-1 have similar activity in people submitted to SG, whereas GLP-1 has the strongest effect after RYGB [36]. The result of this enteric hormone cocktail is an increase in postprandial insulin secretion, acclaimed as the incretin effect [34–36].

We observe higher levels of insulin/C-peptide secretion after C-RYGB, compared to the other three interventions. This may be because all other three interventions achieve a more distal bypass, thus lesser stimulation of K-cells (which are mostly bypassed) and lesser GIP secretion. Instead, all four interventions greatly stimulate the distal L-cells, thus in line with the high GLP-1 levels documented postprandially after all surgical interventions. We hypothesize that GIP is the surplus driver of the augmented insulin/C-peptide secretion documented in C-RYGB, while GLP-1 is likely a potent stimulator in all four procedures.

We also highlight the cross-validation and release of the Gluc4all tool. With a modern interface and an analysis pipeline specifically designed for post-bariatric and non-diabetic cohorts, Gluc4All supports updated CGM metrics [17] along an analysis protocol previously established by our team [15]. Gluc4All allows for custom time windows, and thus event-based alignment. In this study, we cross-validated its performance against manual data analysis and statistical agreement analyses, confirming its robustness and reliability. Gluc4All offers a purpose-built, up-to-date, and user-friendly solution, designed by and for researchers working with high-resolution, physiologically diverse glucose data, but also easily applicable in physicians’ everyday practices.

Challenges to our findings could arise from our study design and/or implementation. While participant selection was unbiased, random, and systematic, the sample size is small, and time since surgery was different between those submitted to RYGB vs hypoabsorptive surgeries, despite all were weight stable at inclusion in the study. Additionally, no formal power calculation was performed. The pre-test hypothesis was strong (i.e., there is accumulating evidence from clinical practice on the challenges posed by the increased glycemic variability after BS) and the study had too many groups and variables for a formal power calculation to yield a reasonable sample size. Furthermore, all *p* values presented were adjusted for multiple testing, which further lowers the test power, and increases the magnitude of the differences required to be detected. Yet, many consistent patterns survive these various statistical challenges and strongly significant differences across groups emerge, which provides reassurance on the methodological approach taken and highlights the strength of the findings herein reported.

The tight inclusion and exclusion criteria allowed us to guarantee the high quality of the dataset. To this day, the isCGM technology used is outdated. We claim, however, that it was the leading technology available at the time of the study, and, most importantly, that we are reassured on the reliability of the data retrieved because the isCGM pattern in the non-operated individuals is well recognized as physiological [17].

It could be argued that having a meal test also on the non-operated participants would provide added insights of the impact of entero-pancreatic hormones on glycemic variability. We believe that would have been redundant as it is well-established that the magnitude of the postprandial entero-pancreatic responses to a meal is much more pronounced after BS due to the gastrointestinal reorganization [36–38].

In summary, we provide the first head-to-head comparison of glycemic variability after four different bariatric procedures by confrontation with non-operated individuals. Glycemic variability is markedly elevated, with an increased risk of postprandial hypoglycemia and associated with modified entero-pancreatic hormone dynamics. Although no cause-effect can be assumed in a cross-sectional study, an overarching link between increased glucose absorption, increased GIP, GLP-1 and insulin secretion and increased glycemic variability is most obvious by comparison of C-RYGB with other procedures and non-operated controls. Most impressively, the anatomical reconstruction of the gut after each type of BS gives rise to a procedure specific glycemic variability pattern.

## Supplementary information

Supplementary Material

## Data Availability

Datasets generated during the current study are not publicly available but are available from the corresponding author on reasonable request.
