# Supplementary material for: Glycemic variability and entero-pancreatic hormones signatures after different bariatric surgery procedures: a cross-sectional study
Source: Int J Obes (Lond). 2025 Aug 9;49(10):2042–50. doi: 10.1038/s41366-025-01865-8 (PMC12532710; doi:10.1038/s41366-025-01865-8)
Supplement: Supplementary file 1 — Supplementary Material [file 41366_2025_1865_MOESM1_ESM.pdf]

Supplementary material to Glycemic variability and entero-pancreatic hormones signatures after different bariatric surgery procedures: A cross-sectional study

Table of Contents

Supplementary Table 1: Inclusion and exclusion criteria. 2
Supplementary Table 2: Timing of peak excursions during the mixed-meal test. 3
Supplementary Table 3: Association between glycemic variability metrics documented by isCGM and during a meal test. 5
Supplementary Table 4: Association between glycemic variability during isCGM and entero-pancreatic hormone profiles during a meal test. 6
Supplementary Table 5: Statistical summary of population characteristics analysis 7
Supplementary Table 6: Statistical summary on data from isCGM. 8
Supplementary Table 7: Statistical summary of entero-pancreatic dynamics following a mixed meal. 10
Supplementary section on Gluc4all validation 12
Supplementary Figure 1. 14
Supplementary Figure 2. 15
Supplementary Figure 3 16
Supplementary Figure 4. 17
Supplementary Table 8 18
STROBE checklist 22
References: 24

Supplementary Table 1: Inclusion and exclusion criteria.

Inclusion criteria	Exclusion criteria
• Adults;<br>• Weight stable (<10% variation) over the previous 6 months;<br>• No history of diabetes (current or former).<br><br>For the operated individuals:<br>• History of one of the following bariatric interventions at least one-year before: C-RYGB, M-RYGB, SADI-S, or BPD-DS;<br>• Weight stable (<10% variation) over the previous 6 months	• HbA1c > 6.5%;<br>• isCGM capture rate <70%<br>• Significant anemia (haemoglobin < 6.5mM / 10.5 g/L);<br>• Known neuropathy or micronutrient / vitamin deficiencies;<br>• Oncological disease;<br>• Glucose altering pharmacotherapy (eg. glucocorticoids, somatostatin analogue treatment, GLP-1 receptor analogue, SGLT2 inhibitor treatment);<br>• Poorly managed thyroid disease (TSH outside of reference range);<br>• Pregnancy;<br>• Renal insufficiency (eGFR < 60ml/min/1.73m2);<br>• Elevation of liver enzymes (> 3x reference range of alanine amino transferase and/or alkaline phosphatases);<br>• Alcohol or substances abuse.<br><br>For the non-operated individuals:<br>• History of any former gastrointestinal surgeries.

Inclusion and exclusion criteria in the study. Abbreviations: C-RYGB – classic Roux-en-Y gastric bypass; M-RYGB – metabolic Roux-en-Y gastric bypass; SADI-S – single anastomosis duodenal-ileal bypass with gastric sleeve; BPS-DS – biliopancreatic diversion with gastric sleeve gastrectomy; isCGM – intermittently scanned continuous glucose monitoring; TSH – thyroid-stimulating hormone; eGFR – estimated glomerular filtration rate.

Supplementary Table 2: Timing of peak excursions during the mixed-meal test.

Variable & group	Peak (timepoint, min)							
	15min	30min	45min	60min	90 min	120min	Missing	Total
**Glucose**								
C-RYGB	1	5	2	-	-	-	0	8
M-RYGB	-	4	2	-	-	-	1	6
SADI-S	-	5	3	-	-	-	0	8
BPD-DS	-	3	3	-	-	-	1	6
Total (n)	1	**17**	10	-	-	-	2	28
**Total amino acids**								
C-RYGB	1	4	3	-	-	-	0	8
M-RYGB	-	-	5	1	-	-	1	6
SADI-S	-	2	4	1	-	-	1	7
BPD-DS	-	3	1	1	-	-	2	5
Total (n)	1	9	**13**	3	-	-	4	26
**Heart rate**								
C-RYGB	1	5	-	1	-	-	1	7
M-RYGB	-	6	1	-	-	-	0	7
SADI-S	-	3	4	-	-	-	1	7
BPD-DS	-	2	2	1	-	-	2	5
Total (n)	1	**16**	7	2	-	-	4	26
**Insulin**								
C-RYGB	-	3	5	-	-	-	0	8
M-RYGB	-	-	5	2	-	-	0	7
SADI-S	-	4	4	0	-	-	0	8
BPD-DS	-	3	2	1	-	-	1	6
Total (n)	-	10	**16**	3	-	-	1	29
**C-peptide**								
C-RYGB	-	4	4	0	-	-	0	8
M-RYGB	-	-	5	2	-	-	0	7
SADI-S	-	1	6	1	-	-	0	8
BPD-DS	-	1	2	3	-	-	1	6
Total (n)	-	6	**17**	6	-	-	1	29
**Glucagon**								
C-RYGB	2	3	1	-	-	2	0	8
M-RYGB	-	-	2	-	2	1	3	4
SADI-S	1	4	1	-	-	-	2	6
BPD-DS	1	1	1	-	1	-	3	4
Total (n)	4	**8**	5	-	3	3	8	22
**Total GIP**								
C-RYGB	2	2	1	-	-	-	3	5
M-RYGB	1	3	1	-	-	-	2	5
SADI-S	1	4	1	2	-	-	-	8
BPD-DS	-	3	2	1	-	-	1	6
Total (n)	4	**12**	5	3	-	-	6	24
**Total GLP-1**								
C-RYGB	1	5	2	-	-	-	0	8
M-RYGB	-	4	3	-	-	-	0	7
SADI-S	1	5	2	-	-	-	0	8
BPD-DS	-	4	2	-	-	-	1	6
Total (n)	2	**18**	9	-	-	-	1	29

Neurotensin								
C-RYGB	2	4	2	-	-	-	0	8
M-RYGB	-	5	2	-	-	-	0	7
SADI-S	-	3	3	-	1	-	1	7
BPD-DS	-	2	-	2	1	1	1	6
Total (n)	2	**14**	7	2	2	1	2	28

Peak timepoints for each hormone during the meal test, organized by surgical group (classic gastric bypass [C-RYGB, n=8], metabolic gastric bypass [M-RYGB, n=7], single anastomosis duodenal-ileal bypass with gastric sleeve [SADI-S, n=8], and biliopancreatic diversion with gastric sleeve [BPD-DS, n=7]). Ambiguous peak values or missing are reported as “missing”.

Supplementary Table 3: Association between glycemic variability metrics documented by isCGM and during a meal test.

		**Glycemic variability during a meal test**						
		MMGR	Peak	Nadir	Glucose tAUC 0-120min	Glucose tAUC 0-peak	TAA tAUC 0-120min	TAA tAUC 0-peak
**Glycemic variability on isCGM**	SD	0.647***	0.605**	-0.194 (ns)	0.724***	0.456*	0.447*	0.455*
	CV	0.689***	0.573**	0.298 (ns)	0.562**	0.066 (ns)	0.405*	0.373*
	Minimum	-0.468*	-0.251 (ns)	0.290 (ns)	-0.305	-0.142	-0.140 (ns)	-0.060 (ns)
	Maximum	0.498**	0.586**	-0.047 (ns)	0.626***	0.503**	0.339 (ns)	0.295 (ns)
	Time < 3.9 mmol/L	0.323 (ns)	0.047 (ns)	-0.279 (ns)	0.060 (ns)	-0.080 (ns)	0.048 (ns)	0.081 (ns)
	TIR	-0.426*	-0.161 (ns)	0.308 (ns)	-0.212 (ns)	-0.008 (ns)	-0.130 (ns)	-0.208 (ns)
	Time > 7.8 mmol/L	0.450*	0.587**	-0.013 (ns)	0.656***	0.511**	0.404*	0.441*
	$LBGI_{FGM}GT$	0.372*	0.023 (ns)	-0.367 (ns)	0.076 (ns)	-0.132	0.028	0.027
	$HBGI_{FGM}GT$	0.400*	0.561**	0.030 (ns)	0.625***	0.505**	0.336	0.388*
	MAG change	0.701***	0.628***	-0.260 (ns)	0.753***	0.383*	0.513**	0.544**
	CONGA1	0.686***	0.631***	-0.217 (ns)	0.743***	0.459*	0.501**	0.498**
	MODD	0.620***	0.596**	-0.212 (ns)	0.622***	0.432*	0.386*	0.390*
	$ADRR_{FGM}GT$	0.721***	0.496**	-0.322 (ns)	0.594**	0.300 (ns)	0.438*	0.382*

Bivariate correlations between glycemic variability metrics obtained on isCGM and during a meal test. The values represent Spearman correlation coefficients. Abbreviations: isCGM – intermittently scanned continuous glucose monitoring; SD – standard deviation; CV – coefficient of variation; TIR – time in range (3.9-7.8 mmol/L; 70-140 mg/dL); $LBGI_{FGM}GT$ – low blood glucose index (adjusted); $HBGI_{FGM}GT$ – high blood glucose index (adjusted); MAG change – mean absolute glucose change; CONGA1 – continuous overlapping net glycemic action; MODD – mean of daily differences; $ADRR_{FGM}GT$ – average daily risk ratio (adjusted); MMGR – minimum-to-maximum glucose ratio; tAUC – total area under the curve; TAA – total amino acids. * p<0.05 ; ** p<0.01 ; *** p<0.001.

Supplementary Table 4: Association between glycemic variability during isCGM and entero-pancreatic hormone profiles during a meal test.

	**MAG change**
**Glucose** tAUC (0'-120')	0.753***
**Glucose** tAUC (0'-30')	0.383*
**TAA** tAUC (0'-120')	0.513**
**TAA** tAUC (0'-45')	0.544**
**Insulin** tAUC (0'-120')	0.530**
**Insulin** tAUC (0'-45')	0.499**
**C-peptide** tAUC (0'-120')	0.656***
**C-peptide** tAUC (0'-45')	0.634***
**Glucagon** tAUC (0'-120')	0.194
**Glucagon** tAUC (0'-30')	-0.117
**GIP** tAUC (0'-120')	0.639***
**GIP** tAUC (0'-30')	0.719***
**GLP-1** tAUC (0'-120')	0.441*
**GLP-1** tAUC (0'-30')	0.633***
**Neurotensin** tAUC (0'-120')	-0.004
**Neurotensin** tAUC (0'-30')	0.301

Bivariate correlations between MAG change and the entero-pancreatic hormone excursions during a meal test. The values represent Spearman correlation coefficients. Abbreviations: isCGM – intermittently scanned continuous glucose monitoring; MAG change – mean absolute glucose change; tAUC – total area under the curve; TAA – total amino acids; GIP – glucose-dependent insulinotropic polypeptide; GLP-1 – glucagon-like peptide-1. * $p<0.05$; ** $p<0.01$; *** $p<0.001$.

Supplementary Table 5: Statistical summary of population characteristics analysis.

Variable	Normal distribution (Y/N/NA)	Log transformed (Y/N/NA)	Test used	Comparisons (corrected *p* values)									
				C-M	C-S	C-D	M-S	M-D	S-D	X-C	X-M	X-S	X-D
Sex	NA	NA	Fisher's	-									
Age	Y	N	ANOVA	-	-	-	-	-	-	-	-	-	-
Weight	Y	N	ANOVA	-	-	-	-	-	-	-	-	-	-
BMI	Y	N	ANOVA	-	-	-	-	-	-	-	-	-	-
eA1c	Y	N	ANOVA	-	-	-	-	-	-	-			
A1c	N	Y	ANOVA	-	**	**	-	-	-				
HOMA2-B	N	N	K-W	-	-	-	-	-	-				
HOMA2-IR	N	N	K-W	-	-	-	-	-	-				
Time since surgery	Y	N	ANOVA	-	**	**	*	*	-				
Weight pre op	Y	N	ANOVA	-	-	*	*	**	-				
BMI pre op	N	Y	ANOVA	-	***	***	***	***	-				
%EBMIL	N	N	K-W	-	-	-	-	-	-				
%TWL	Y	N	ANOVA	-	**	***	*	**	-				
A1c pre op	Y	N	ANOVA	-	-	-	-	-	-				

Statistical summary on the demographic and anthropometric features of the study participants distributed per study group (classic Roux-en-Y gastric bypass [C, n=8], metabolic Roux-en-Y gastric bypass [M, n=7], single anastomosis duodenal-ileal bypass with gastric sleeve [S, n=8], biliopancreatic diversion with gastric sleeve [D, n=7] and non-operated matched individuals [X, n=8]. Abbreviations: Fisher's – Fisher's exact test; ANOVA – Ordinary one-way ANOVA with Tukey's multiple comparisons test (*p* values adjusted for testing between surgeries [nr of tests = 6] and with non-operated population [nr of tests = 4]); K-W – Kruskal-Wallis test with Dunn's multiple comparisons test (*p* values adjusted for the number of pairs); HOMA2-B – homeostasis model assessment of β-cell function; HOMA2-IR – homeostasis model assessment of insulin resistance; eA1c – estimated percentage of glycated haemoglobin; %EBMIL – percentage of excess body mass index loss; %TWL – percentage of total weight loss. * $p<0.05$; ** $p<0.01$; *** $p<0.001$.

Supplementary Table 6: Statistical summary on data from isCGM.

Variable	Normal distribution (Y/N/NA)	Log transformed (Y/N/NA)	Test used	Comparisons (corrected *p* values)									
				C-M	C-S	C-D	M-S	M-D	S-D	X-C	X-M	X-S	X-D
Duration (days)	N	N	K-W	-	-	-	-	-	-	-	*	**	-
Data capture rate (%)	N	N	K-W	-	-	-	-	-	-	-	-	-	-
TIR (%)	N	N	K-W	-	-	-	-	-	-	**	*	-	-
Time < 3.9 mmol/L (%)	N	NA	K-W	-	-	-	-	-	-	*	-	-	-
Time < 3.0 mmol/L (%)	N	NA	K-W	-	-	-	-	-	-	*	-	-	-
Time > 7.8 mmol/L (%)	N	NA	K-W	-	-	*	-	-	-	**	*	-	-
P10 (mmol/L)	Y	N	ANOVA	-	-	-	-	-	-	**	*	*	*
P25 (mmol/L)	Y	N	ANOVA	-	-	-	-	-	-	*	*	*	-
P50 (mmol/L)	Y	N	ANOVA	-	-	-	-	-	-	-	-	-	-
P75 (mmol/L)	Y	N	ANOVA	-	-	-	-	-	-	-	-	-	-
P90 (mmol/L)	Y	N	ANOVA	-	-	-	-	-	-	*	**	-	-
Maximum (mmol/L)	Y	N	ANOVA	-	*	**	-	*	-	***	**	-	-
$\text{LBGI}_{\text{FGM}}\text{GT}$	N	NA	K-W	-	-	-	-	-	-	*	-	-	-
$\text{HBGI}_{\text{FGM}}\text{GT}$	N	NA	K-W	-	-	-	-	-	-	*	-	-	-
MAG change (mmol/L × h^{-1})	Y	N	ANOVA	-	***	**	*	*	-	***	***	**	*
CONGA1	N	Y	ANOVA	-	**	**	*	*	-	***	***	**	**
MODD	Y	N	ANOVA	-	**	***	*	**	-	***	***	*	-
$\text{ADRR}_{\text{FGM}}\text{GT}$	Y	N	ANOVA	-	*	**	-	-	-	***	**	-	-
Mean	Y	N	ANOVA	-	-	-	-	-	-	-	-	-	-
SD	N	N	K-W	-	*	**	-	-	-	***	***	-	-
CV (%)	N	N	K-W	-	*	**	-	-	-	***	***	-	-

Statistical summary on the data retrieved using isCGM. Study participants are distributed per study group (classic gastric bypass [C, n=8], metabolic gastric bypass [M, n=7], single anastomosis duodenal-ileal bypass with gastric sleeve [S, n=8], biliopancreatic diversion with gastric sleeve [D, n=7] and non-operated matched individuals [X, n=8]. Abbreviations: K-W – Kruskal-Wallis test with Dunn's multiple comparisons test (*p* values adjusted for testing between surgeries [nr of tests = 6] and with non-operated population [nr of tests = 4]); ANOVA – Ordinary one-way ANOVA with Tukey's multiple comparisons test (*p* values adjusted for the number of pairs); TIR – time in range (3.9-7.8 mmol/L; 70-140 mg/dL); P – percentile; $LBGI_{FGM}GT$ – low blood glucose index (adjusted); $HBGI_{FGM}GT$ – high blood glucose index (adjusted); MAG change – mean absolute glucose change; CONGA1 – continuous overlapping net glycemic action; MODD – mean of daily differences; $ADRR_{FGM}GT$ – average daily risk ratio (adjusted); SD – standard deviation; CV – coefficient of variation. * $p<0.05$; ** $p<0.01$; *** $p<0.001$.

Supplementary Table 7: Statistical summary of entero-pancreatic dynamics following a mixed meal.

Variable	Normal distribution (Y/N/NA)	Log transformed (Y/N/NA)	Test used	Comparisons (corrected p values)					
				C-M	C-S	C-D	M-S	M-D	S-D
**Glucose**									
Fasted	Y	N	ANOVA	-	-	-	-	-	-
Peak	N	N	K-W	-	-	**	-	-	-
Nadir	Y	N	ANOVA	-	*	-	-	-	-
MMGR	Y	N	ANOVA	*	***	***	-	-	-
tAUC (0’-30’)	Y	N	ANOVA	-	***	***	*	***	-
tAUC (0’-120’)	Y	N	ANOVA	-	-	-	-	*	-
**TAA**									
Fasted	Y	N	ANOVA	-	*	**	-	-	-
tAUC (0’-45’)	Y	N	ANOVA	-	-	***	-	*	*
tAUC (0’-120’)	N	N	K-W	-	-	**	-	*	-
**Heart rate**									
Fasted	N	Y	ANOVA	-	-	-	-	-	-
tAUC (0’-30’)	N	N	K-W	-	-	-	-	-	-
tAUC (0’-120’)	N	N	K-W	-	-	-	-	-	-
**Insulin**									
Fasted	N	N	K-W	-	-	-	-	-	-
tAUC (0’-45’)	N	N	K-W	*	-	**	-	-	-
tAUC (0’-120’)	Y	N	ANOVA	*	***	***	-	-	-
**C-peptide**									
Fasted	N	N	K-W	-	-	*	-	-	-
tAUC (0’-45’)	N	N	K-W	-	*	***	-	-	-
tAUC (0’-120’)	N	N	K-W	-	*	**	-	-	-
**Glucagon**									
tAUC (0’-30’)	Y	N	ANOVA	-	-	-	-	-	-

tAUC (0'-120')	Y	N	ANOVA	-	-	-	-	-	-
**Total GIP**									
tAUC (0'-30')	N	Y	ANOVA	-	***	***	-	-	-
tAUC (0'-120')	N	Y	ANOVA	-	**	**	-	-	-
**Total GLP-1**									
tAUC (0'-30')	N	Y	ANOVA	-	-	-	-	-	-
tAUC (0'-120')	N	Y	ANOVA	-	-	-	-	*	-
**Neurotensin**									
tAUC (0'-30')	N	N	K-W	-	-	-	-	-	-
tAUC (0'-120')	N	N	K-W	-	-	-	-	-	-

Statistical summary on the data retrieved during the meal test on the entero-pancreatic hormone profile distributed per study group (classic Roux-en-Y gastric bypass [C, n=8], metabolic Roux-en-Y gastric bypass [M, n=7], single anastomosis duodenal-ileal bypass with gastric sleeve [S, n=8] and biliopancreatic diversion with gastric sleeve [D, n=7]). Data are presented as mean [95% confidence interval of the mean] or median (interquartile range). Abbreviations: K-W – Kruskal-Wallis test with Dunn's multiple comparisons test (*p* values adjusted for testing between surgeries [nr of tests = 6] and with non-operated population [nr of tests = 4]); ANOVA – Ordinary one-way ANOVA with Tukey's multiple comparisons test (*p* values adjusted for the number of pairs); MMGR – minimum-to-maximum glucose ratio; tAUC – total area under the curve; TAA – total amino acids; GIP – glucose-dependent insulinotropic polypeptide; GLP-1 – glucagon-like peptide-1. * $p<0.05$; ** $p<0.01$; *** $p<0.001$.

Supplementary section on Gluc4all validation

We developed an automated tool, Gluc4all, using Visual Basic for Applications to optimize and automate continuous glucose monitoring (CGM) data analysis, using targeted metrics. Version 1.0.0 is particularly relevant for populations without diabetes on whom focusing on glycemic variability is of particular relevance, eg. individuals without glucose intolerance, previously submitted to bariatric surgery. We previously described these metrics extensively (1). The goal of creating this automated spreadsheet was to develop a tool that can be easily used by researchers and clinicians, without requiring an internet connection or prior installation, and to make it widely available (**Supplementary Figures 1-4**). Cross-validation information is provided in **Supplementary Tables 2 and 3**.

For a given CGM data file, and a certain time period (determined by the user), Gluc4all version 1.0.0 returns:

- CGM monitoring duration;
- Valid readings (number of consecutive readings, without missing information);
- Data capture rate;
- Minimum, percentiles 10, 25, 50, 75 and 90, and maximum;
- Time below 54 mg/dL / 3.0 mmol/L (level 2 hypoglycemia);
- Time below 70 mg/dL / 3.9 mmol/L (level 1 hypoglycemia);
- Time in range (70-140 mg/dL / 3.9-7.8 mmol/L);
- Time above 140 mg/dL / 7.8 mmol/L (hyperglycemia);
- Mean glucose value, standard deviation (SD), and coefficient of variation (CV);
- Low blood glucose index (adjusted for individuals without diabetes, $LBGI_{FGM}GT$);
- High blood glucose index (adjusted for individuals without diabetes, $HBGI_{FGM}GT$);
- Average daily risk ratio (adjusted for individuals without diabetes, $ADRR_{FGM}GT$);
- Mean absolute glucose change (MAG change);
- Continuous overlapping net glycemic action (CONGA1);
- Mean of daily differences (MODD).

The tool automatically identifies data missing and accounts for that when calculating, for instance, capture rate, time in range, and MAG change, without data interpolation. Please refer to our previous publication (1) for a detailed description of each metric above listed. Gluc4all version 1.0.0 display is shown at **Supplementary Figures 1-4**. Gluc4all version 1.0.0 supports analysis of .xls files, with an option for simultaneous group analysis of various date files. It analysis CGM data from a time period defined by the user, thus enabling the analysis of the totality of a data file or only a time segment of it.

Gluc4all version 1.0.0. is only operational with Abbott FreeStyle Libre® generated output in mg/dL (models Libre 1, Libre 2 and LibrePro), but posterior updates (to be made available in the same domain: https://umib.icbas.up.pt/) will cover other units and equipments.

All in all, Gluc4all version 1.0.0 is an intuitive, user-friendly, and hereby made widely available tool, that is free, and that paves the way for systematic and universal CGM data analysis in patient populations without diabetes.

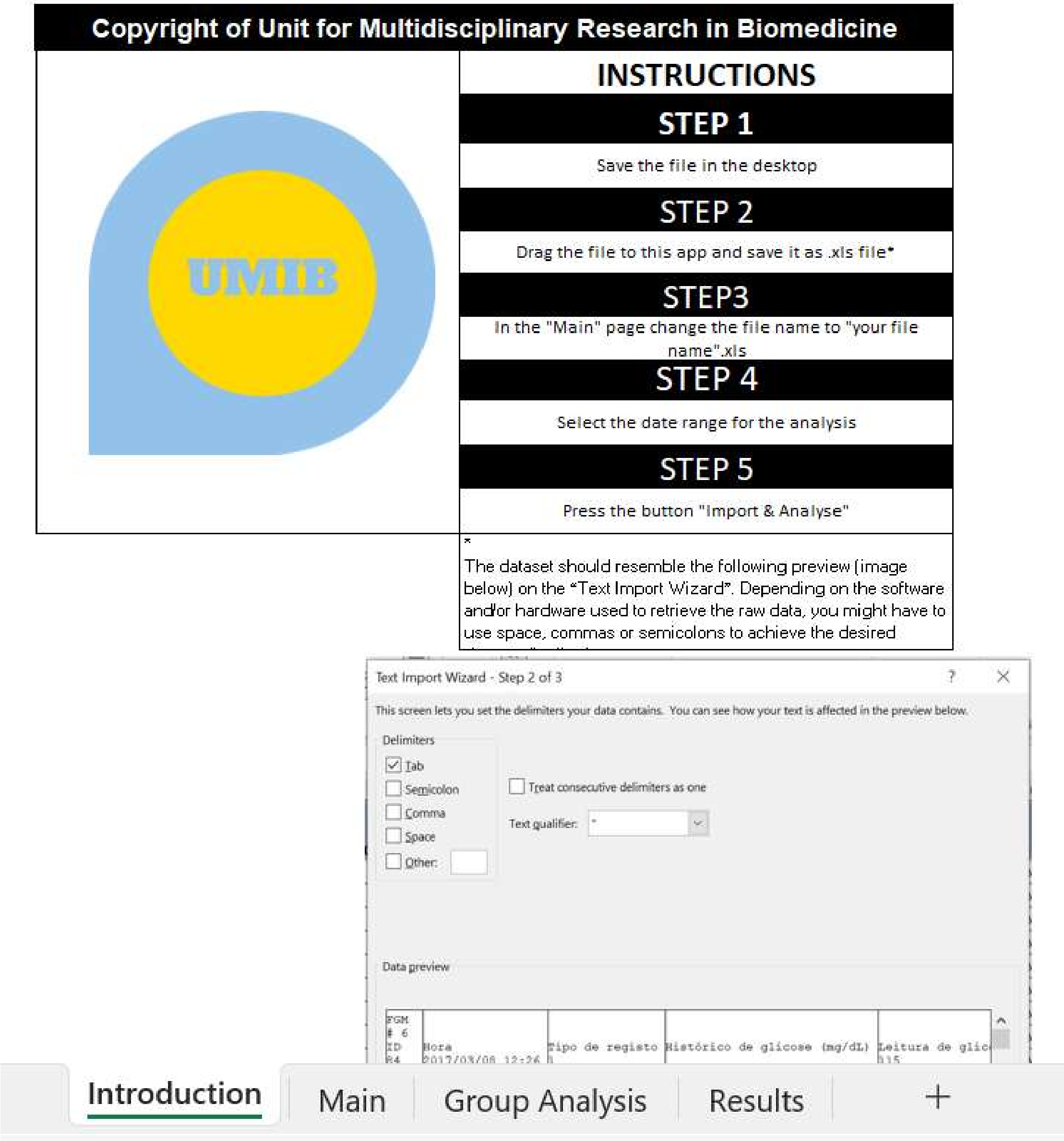

Supplementary Figure 1. Gluc4all front screen display. The frontpage of Gluc4all 1.0.0. contains a step-by-step guide with instructions on how to use the worksheet.

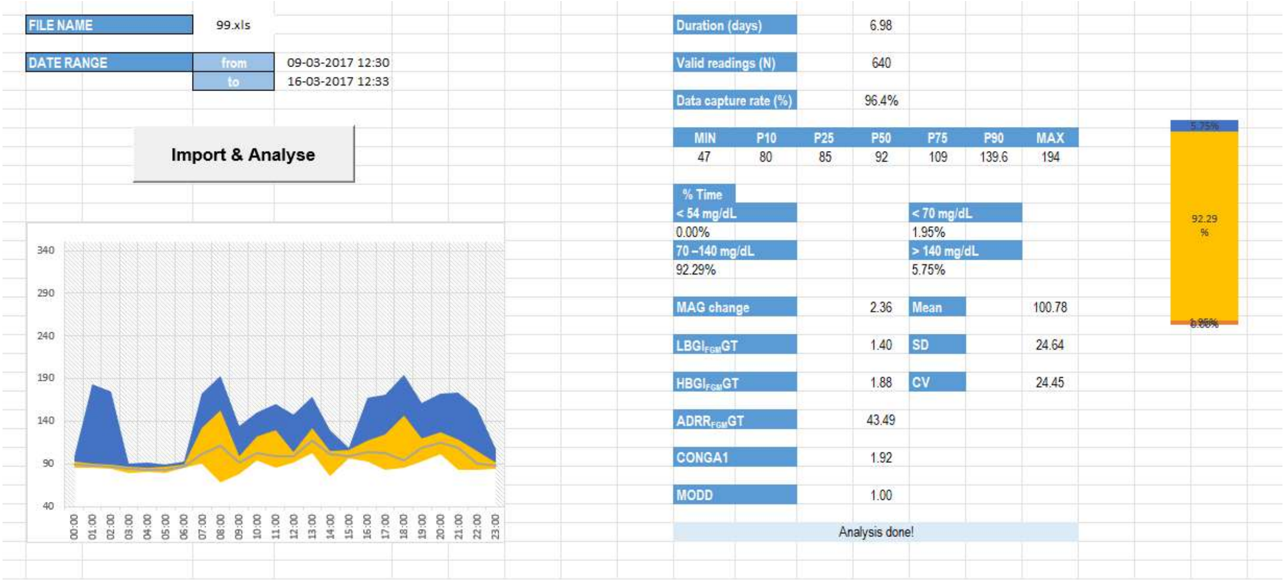

Supplementary Figure 2. Gluc4all display of data analysis. The tab named "Main" allows the import and analysis of a single CGM data file. Firstly, you have to type in the name of the file from which data will be imported and the time period from which data should be imported. Afterwards, you have to press "Import & Analyse", which will return automatically all the glucose metrics previously mentioned. It also displays an average daily glucose profile. Please note that the results displayed in this example were generated after a fictitious dataset. Abbreviations: Min – minimum; P – percentile; Max – maximum; MAG change – mean absolute glucose change; $LBGI_{FGM}GT$ – low blood glucose index (adjusted); $HBGI_{FGM}GT$ – high blood glucose index (adjusted); $ADRR_{FGM}GT$ – average daily risk ratio (adjusted); CONGA1 – continuous overlapping net glycemic action; MODD – mean of daily differences; SD – standard deviation; CV – coefficient of variation.

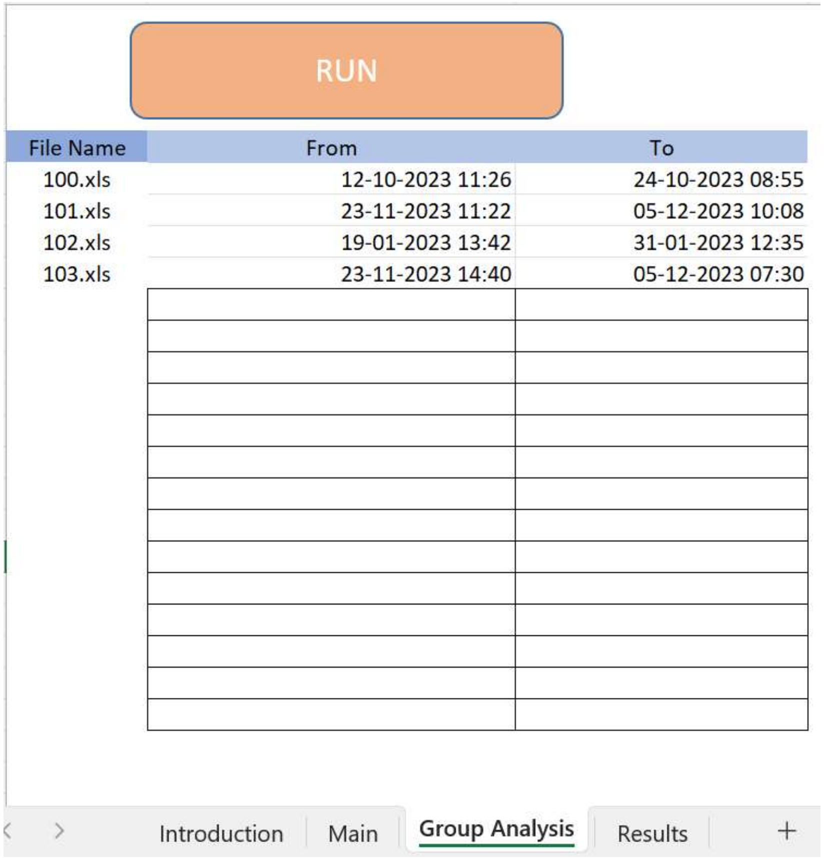

File Name	From	To
100.xls	12-10-2023 11:26	24-10-2023 08:55
101.xls	23-11-2023 11:22	05-12-2023 10:08
102.xls	19-01-2023 13:42	31-01-2023 12:35
103.xls	23-11-2023 14:40	05-12-2023 07:30

Supplementary Figure 3. Gluc4all display of group data analysis input. Simultaneous analysis of multiple data files is possible using the "Group Analysis" tab. This requires listing the different file names, and timestamp "from" and "to" which each file should be analyzed. Once "Run" is pressed, the results of such analysis will be displayed in the following tab ("Results", Supplementary Figure 4).

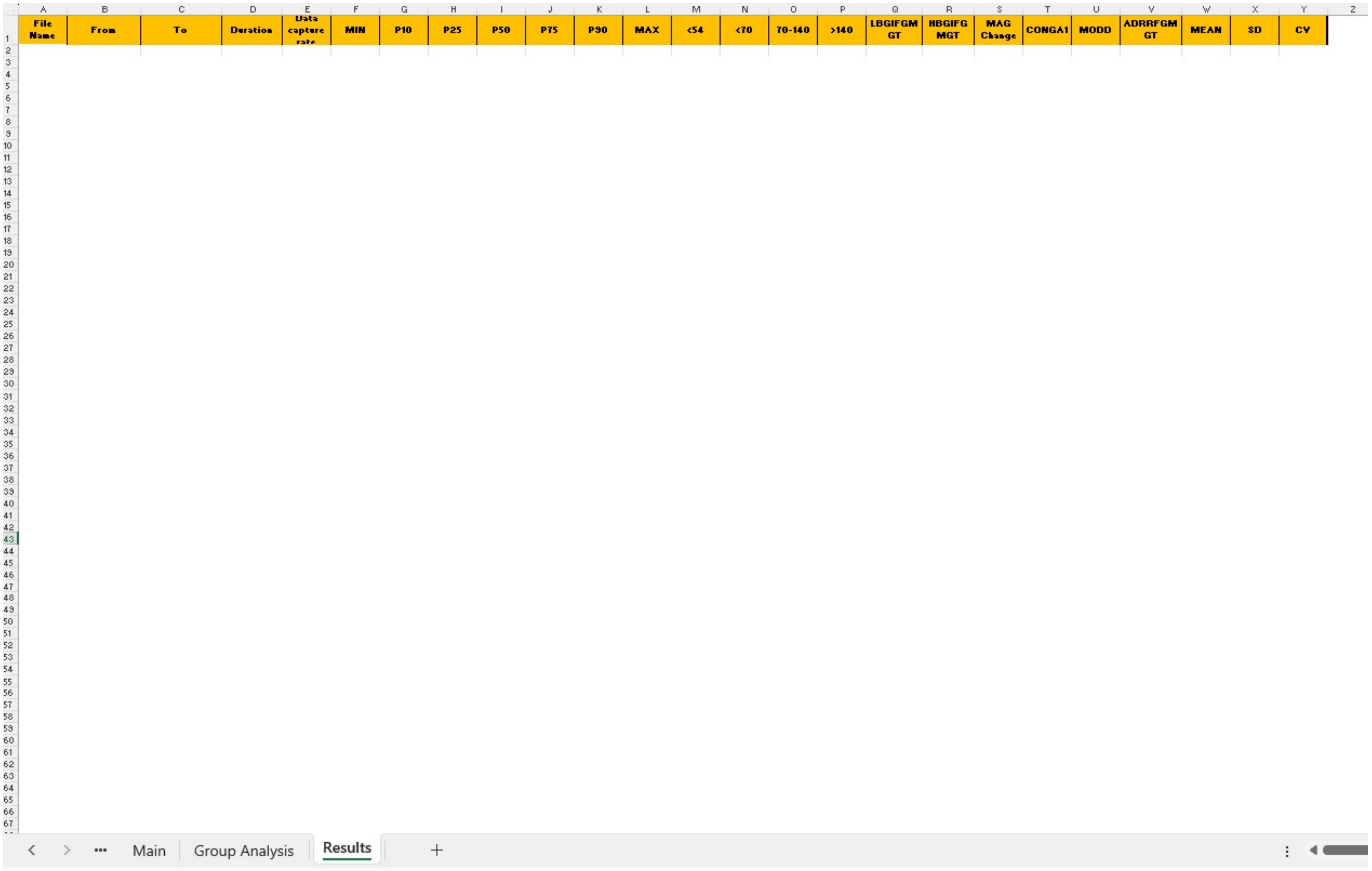

Supplementary Figure 4. Gluc4all display of group data analysis output. The results from multiple files simultaneous analysis are displayed in the last tab, in the format of a table, where a line is generated per each file analyzed. Abbreviations: Min – minimum; P – percentile; Max – maximum; $LBGI_{FGM}GT$ – low blood glucose index (adjusted); $HBGI_{FGM}GT$ – high blood glucose index (adjusted); MAG change – mean absolute glucose change; CONGA1 – continuous overlapping net glycemic action; MODD – mean of daily differences; $ADRR_{FGM}GT$ – average daily risk ratio (adjusted); SD – standard deviation; CV – coefficient of variation.

Supplementary Table 8. Cross-validation between manual and the Gluc4all 1.0.0. automated tool analysis, through percentage difference and T-test (1 of 2).

File / Percentage Difference (%)	**Duration**	**Data capture rate**	**Minimum**	**P10**	**P25**	**P50**	**P75**	**P90**	**Maximum**	**Mean**	**SD**	**CV**
#1	0.00	0.00	0.00	0.00	0.00	0.00	0.00	0.00	0.00	0.00	0.00	0.00
#2	0.00	0.00	0.00	0.00	0.00	0.00	0.00	0.00	0.00	0.00	0.00	0.00
#3	0.00	0.00	0.00	0.00	0.00	0.00	0.00	0.00	0.00	0.00	0.00	0.00
#4	0.00	0.00	0.00	0.00	0.00	0.00	0.00	0.00	0.00	0.00	0.00	0.00
#5	0.00	0.00	0.00	0.00	0.00	0.00	0.00	0.00	0.00	0.00	0.00	0.00
#6	0.00	0.00	0.00	0.00	0.00	0.00	0.00	0.00	0.00	0.00	0.00	0.00
#7	0.00	0.00	0.00	0.00	0.00	0.00	0.00	0.00	0.00	0.00	0.00	0.00
#8	0.00	0.00	0.00	0.00	0.00	0.00	0.23	0.00	0.00	0.00	0.00	0.00
#9	0.00	0.00	0.00	0.00	0.00	0.00	0.00	0.00	0.00	0.00	0.00	0.00
#10	0.00	0.00	0.00	0.00	0.00	0.00	0.00	0.00	0.00	0.00	0.00	0.00
#11	0.00	0.00	0.00	0.00	0.00	0.00	0.00	0.00	0.00	0.00	0.00	0.00
#12	0.00	0.00	0.00	0.00	0.00	0.00	0.00	0.00	0.00	0.00	0.00	0.00
#13	0.00	0.00	0.00	0.00	0.00	0.00	0.25	0.00	0.00	0.00	0.00	0.00
#14	0.00	0.00	0.00	0.00	0.00	0.00	0.00	0.00	0.00	0.00	0.00	0.00
#15	0.00	0.00	0.00	0.00	0.00	0.00	0.00	0.00	0.00	0.01	0.04	0.00
#16	0.00	0.00	0.00	0.00	0.00	0.00	0.00	0.00	0.00	0.00	0.00	0.00
#17	0.00	0.00	0.00	0.00	0.00	0.00	0.00	0.00	0.00	0.00	0.00	0.00
#18	0.00	0.00	0.00	0.00	0.00	0.00	0.00	0.00	0.00	0.00	0.00	0.00
#19	0.00	0.00	0.00	0.00	0.00	0.00	0.00	0.00	0.00	0.00	0.00	0.00
#20	0.00	0.00	0.00	0.00	0.00	0.00	0.00	0.00	0.00	0.00	0.00	0.00
#21	0.00	0.00	0.00	0.00	0.00	0.00	0.00	0.00	0.00	0.00	0.00	0.00
#22	0.00	0.00	0.00	0.00	0.00	0.00	0.00	0.00	0.00	0.00	0.00	0.00
#23	0.00	0.00	0.00	0.00	0.00	0.00	0.00	0.00	0.00	0.00	0.00	0.00

#24	0.00	0.00	0.00	0.00	0.00	0.00	0.00	0.00	0.00	0.00	0.00	0.00
#25	0.00	0.00	0.00	0.00	0.00	0.00	0.00	0.00	0.00	0.00	0.00	0.00
#26	0.00	0.00	0.00	0.00	0.00	0.00	0.00	0.00	0.00	0.00	0.00	0.00
#27	0.00	0.00	0.00	0.00	0.00	0.00	0.00	0.00	0.00	0.00	0.00	0.00
#28	0.00	0.00	0.00	0.00	0.00	0.00	0.00	0.00	0.00	0.00	0.00	0.00
#29	0.00	0.00	0.00	0.00	0.00	0.00	0.00	0.00	0.00	0.00	0.00	0.00
#30	0.00	0.00	0.00	0.00	0.00	0.00	0.00	0.00	0.00	0.00	0.00	0.00
#31	0.00	0.00	0.00	0.00	0.00	0.00	0.00	0.00	0.00	0.00	0.00	0.00
#32	0.00	0.00	0.00	0.00	0.00	0.00	0.00	0.00	0.00	0.00	0.00	0.00
#33	0.00	0.00	0.00	0.00	0.00	0.00	0.00	0.00	0.00	0.00	0.00	0.00
#34	0.00	0.00	0.00	0.00	0.00	0.00	0.00	0.00	0.00	0.00	0.00	0.00
#35	0.08	0.00	0.00	0.00	0.00	0.00	0.00	0.00	0.00	0.00	0.00	0.00
#36	0.00	0.00	0.00	0.00	0.00	0.00	0.00	0.00	0.00	0.00	0.00	0.00
#37	0.00	0.00	0.00	0.00	0.00	0.00	0.00	0.00	0.00	0.00	0.00	0.00
#38	0.00	0.00	0.00	0.00	0.00	0.00	0.00	0.00	0.00	0.00	0.00	0.00
***p* value**	*0.21*	*1.00*	*1.00*	*1.00*	*1.00*	*1.00*	*0.16*	*1.00*	*1.00*	*0.32*	*0.32*	*1.00*

(*Table caption below*)

Supplementary Table 8 (continued). Cross-validation between manual and the Gluc4all 1.0.0. automated tool analysis, through percentage difference and T-test (2 of 2).

File / Percentage Difference (%)	**Time< 3.0 mmol/L / 54 mg/dL**	**Time< 3.9 mmol/L / 70 mg/dL**	**TIR**	**Time> 7.8 mmol/L / 140 mg/dL**	**$LBGI_{FGM}$ GT**	**$HBGI_{FGM}$ GT**	**MAG Change**	**CONGA1**	**MODD**	**$ADRR_{FGM}$ GT**
**#1**	0.00	0.00	0.00	0.00	0.00	0.00	0.00	0.00	0.00	0.00
**#2**	0.00	0.00	0.00	0.00	0.00	0.00	0.00	0.00	0.00	0.00
**#3**	0.00	0.00	0.00	0.00	0.00	0.00	0.00	0.00	0.00	0.00
**#4**	0.00	0.00	0.00	0.00	0.00	0.00	0.00	0.75	0.00	0.00
**#5**	0.00	0.00	0.00	0.00	0.00	0.00	0.00	0.00	0.00	0.00
**#6**	0.00	0.00	0.00	0.00	0.00	0.00	0.00	0.00	0.00	0.00
**#7**	0.00	0.00	0.00	0.00	0.00	0.00	0.00	0.00	0.00	0.00
**#8**	0.00	0.00	0.00	0.00	0.00	0.00	0.00	0.00	0.00	0.00
**#9**	0.00	0.00	0.00	0.00	0.00	0.00	0.00	0.00	0.00	0.00
**#10**	0.00	0.00	0.00	0.00	0.00	0.00	0.00	0.00	0.00	0.00
**#11**	0.00	0.00	0.00	0.00	0.00	0.00	0.00	0.00	0.00	0.00
**#12**	0.00	0.00	0.00	0.00	0.00	0.00	0.00	0.00	0.00	0.00
**#13**	0.00	0.00	0.00	0.00	0.00	0.00	0.00	0.00	0.00	0.00
**#14**	0.00	0.00	0.00	0.00	0.00	0.00	0.00	0.00	0.00	0.00
**#15**	0.00	0.00	0.00	0.00	0.00	0.00	0.00	0.00	0.00	0.00
**#16**	0.00	0.00	0.00	0.00	0.00	0.00	0.00	0.00	0.00	0.00
**#17**	0.00	0.00	0.00	0.00	0.00	0.00	0.00	0.00	0.00	0.00
**#18**	0.00	0.00	0.00	0.00	0.00	0.00	0.00	0.00	0.00	0.00
**#19**	0.00	0.00	0.00	0.00	0.00	0.00	0.00	0.00	0.00	0.00
**#20**	0.00	0.00	0.00	0.00	0.00	0.00	0.00	0.00	0.00	0.00
**#21**	0.00	0.00	0.00	0.00	0.00	0.00	0.00	0.00	0.00	0.00
**#22**	0.00	0.00	0.00	0.00	0.00	0.00	0.00	0.00	0.00	0.00
**#23**	0.00	0.00	0.00	0.00	0.00	0.00	0.00	0.00	0.00	0.00
**#24**	0.00	0.00	0.00	0.00	0.00	0.00	0.00	0.00	0.00	0.00

**#25**	0.00	0.00	0.00	0.00	0.00	0.00	0.00	0.00	0.00	0.00
**#26**	0.00	0.00	0.00	0.00	0.00	0.00	0.00	0.00	0.00	0.00
**#27**	0.00	0.00	0.00	0.00	0.00	0.00	0.00	0.00	0.00	1.25
**#28**	0.00	0.00	0.00	0.00	0.00	0.00	0.00	0.00	0.00	2.70
**#29**	0.00	0.00	0.00	0.00	0.00	0.00	0.00	0.00	0.00	0.00
**#30**	0.00	0.00	0.00	0.00	0.00	0.00	0.00	0.00	0.00	0.00
**#31**	0.00	0.00	0.00	0.00	0.00	0.00	0.00	0.00	0.00	0.00
**#32**	0.00	0.00	0.00	0.00	0.00	0.00	0.00	0.00	0.00	0.00
**#33**	0.00	0.00	0.00	0.00	0.00	0.00	0.00	0.00	0.00	0.00
**#34**	0.00	0.00	0.00	0.00	0.00	0.00	0.00	0.00	0.00	0.00
**#35**	0.00	0.00	0.00	0.00	0.00	0.00	0.00	0.00	0.00	0.00
**#36**	0.00	0.00	0.00	0.00	0.00	0.00	0.00	0.00	0.00	0.00
**#37**	0.00	0.00	0.00	0.00	0.00	0.00	0.00	0.00	0.00	0.00
**#38**	0.00	0.00	0.00	0.00	0.00	0.00	0.00	0.00	0.00	0.00
***p* value**	*1.00*	*1.00*	*1.00*	*1.00*	*1.00*	*1.00*	*1.00*	*1.00*	*0.32*	*1.00*

Data from intermittently scanned continuous glucose monitoring (isCGM) of all the study participants (N=38). *p* values presented refer to one sample T-tests against 0. Abbreviations: P – percentile; SD – standard deviation; CV – coefficient of variation; TIR – time in range (3.9-7.8 mmol/L; 70-140 mg/dL); $LBGI_{FGM}GT$ – low blood glucose index (adjusted); $HBGI_{FGM}GT$ – high blood glucose index (adjusted); MAG change – mean absolute glucose change; CONGA1 – continuous overlapping net glycemic action; MODD – mean of daily differences; $ADRR_{FGM}GT$ – average daily risk ratio (adjusted).

STROBE Statement—checklist of items that should be included in reports of observational studies

	Item No	Recommendation	Page number
**Title and abstract**	1	(*a*) Indicate the study's design with a commonly used term in the title or the abstract	1
		(*b*) Provide in the abstract an informative and balanced summary of what was done and what was found	2
**Introduction**			
Background/rationale	2	Explain the scientific background and rationale for the investigation being reported	4
Objectives	3	State specific objectives, including any prespecified hypotheses	4
**Methods**			
Study design	4	Present key elements of study design early in the paper	5
Setting	5	Describe the setting, locations, and relevant dates, including periods of recruitment, exposure, follow-up, and data collection	5-6
Participants	6	(*a*) *Cohort study*—Give the eligibility criteria, and the sources and methods of selection of participants. Describe methods of follow-up<br>*Case-control study*—Give the eligibility criteria, and the sources and methods of case ascertainment and control selection. Give the rationale for the choice of cases and controls<br>*Cross-sectional study*—Give the eligibility criteria, and the sources and methods of selection of participants	5
		(*b*) *Cohort study*—For matched studies, give matching criteria and number of exposed and unexposed<br>*Case-control study*—For matched studies, give matching criteria and the number of controls per case	5
Variables	7	Clearly define all outcomes, exposures, predictors, potential confounders, and effect modifiers. Give diagnostic criteria, if applicable	7
Data sources/ measurement	8*	For each variable of interest, give sources of data and details of methods of assessment (measurement). Describe comparability of assessment methods if there is more than one group	7
Bias	9	Describe any efforts to address potential sources of bias	8-9
Study size	10	Explain how the study size was arrived at	17
Quantitative variables	11	Explain how quantitative variables were handled in the analyses. If applicable, describe which groupings were chosen and why	8
Statistical methods	12	(*a*) Describe all statistical methods, including those used to control for confounding	8
		(*b*) Describe any methods used to examine subgroups and interactions	8
		(*c*) Explain how missing data were addressed	8
		(*d*) *Cohort study*—If applicable, explain how loss to follow-up was addressed<br>*Case-control study*—If applicable, explain how matching of cases and controls was addressed<br>*Cross-sectional study*—If applicable, describe analytical methods taking account of sampling strategy	8
		(*e*) Describe any sensitivity analyses	-

Continued on next page

**Results**			
Participants	13*	(a) Report numbers of individuals at each stage of study—eg numbers potentially eligible, examined for eligibility, confirmed eligible, included in the study, completing follow-up, and analysed	9
		(b) Give reasons for non-participation at each stage	-
		(c) Consider use of a flow diagram	-
Descriptive data	14*	(a) Give characteristics of study participants (eg demographic, clinical, social) and information on exposures and potential confounders	9
		(b) Indicate number of participants with missing data for each variable of interest	11
		(c) *Cohort study*—Summarise follow-up time (eg, average and total amount)	-
Outcome data	15*	*Cohort study*—Report numbers of outcome events or summary measures over time	-
		Case-control study—Report numbers in each exposure category, or summary measures of exposure	-
		Cross-sectional study—Report numbers of outcome events or summary measures	*9-11*
	16	(*a*) Give unadjusted estimates and, if applicable, confounder-adjusted estimates and their precision (eg, 95% confidence interval). Make clear which confounders were adjusted for and why they were included	9-11
		(*b*) Report category boundaries when continuous variables were categorized	9-11
		(*c*) If relevant, consider translating estimates of relative risk into absolute risk for a meaningful time period	-
Other analyses	17	Report other analyses done—eg analyses of subgroups and interactions, and sensitivity analyses	-
**Discussion**			
Key results	18	Summarise key results with reference to study objectives	11
Limitations	19	Discuss limitations of the study, taking into account sources of potential bias or imprecision. Discuss both direction and magnitude of any potential bias	17-18
Interpretation	20	Give a cautious overall interpretation of results considering objectives, limitations, multiplicity of analyses, results from similar studies, and other relevant evidence	11-18
Generalisability	21	Discuss the generalisability (external validity) of the study results	17-18
**Other information**			
Funding	22	Give the source of funding and the role of the funders for the present study and, if applicable, for the original study on which the present article is based	19

*Give information separately for cases and controls in case-control studies and, if applicable, for exposed and unexposed groups in cohort and cross-sectional studies.

Note: An Explanation and Elaboration article discusses each checklist item and gives methodological background and published examples of transparent reporting. The STROBE checklist is best used in conjunction with this article (freely available on the Web sites of PLoS Medicine at http://www.plosmedicine.org/, Annals of Internal Medicine at http://www.annals.org/, and Epidemiology at http://www.epidem.com/). Information on the STROBE Initiative is available at www.strobe-statement.org.

References:

1. Lobato CB, Pereira SS, Guimarães M, Morais T, Oliveira P, de Carvalho JPM, Nora M, Monteiro MP. Use of flash glucose monitoring for post-bariatric hypoglycaemia diagnosis and management. Scientific Reports 2020;10:11061
